# Biomarkers in Heart Failure with Preserved Ejection Fraction: A Perpetually Evolving Frontier

**DOI:** 10.3390/jcm13164627

**Published:** 2024-08-07

**Authors:** Ana-Maria Vrabie, Stefan Totolici, Caterina Delcea, Elisabeta Badila

**Affiliations:** 1 Cardio-Thoracic Pathology Department, “Carol Davila” University of Medicine and Pharmacy, 050474 Bucharest, Romania; stefan.totolici@drd.umfcd.ro (S.T.); caterina.delcea@umfcd.ro (C.D.); elisabeta.badila@umfcd.ro (E.B.); 2 Cardiology Department, Colentina Clinical Hospital, 020125 Bucharest, Romania

**Keywords:** heart failure with preserved ejection fraction, HFpEF, biomarkers, natriuretic peptides, troponins, diagnostic biomarkers, prognostic biomarkers

## Abstract

Heart failure with preserved ejection fraction (HFpEF) represents a complex clinical syndrome, often very difficult to diagnose using the available tools. As the global burden of this disease is constantly growing, surpassing the prevalence of heart failure with reduced ejection fraction, during the last few years, efforts have focused on optimizing the diagnostic and prognostic pathways using an immense panel of circulating biomarkers. After the paradigm of HFpEF development emerged more than 10 years ago, suggesting the impact of multiple comorbidities on myocardial structure and function, several phenotypes of HFpEF have been characterized, with an attempt to find an ideal biomarker for each distinct pathophysiological pathway. Acknowledging the limitations of natriuretic peptides, hundreds of potential biomarkers have been evaluated, some of them demonstrating encouraging results. Among these, soluble suppression of tumorigenesis-2 reflecting myocardial remodeling, growth differentiation factor 15 as a marker of inflammation and albuminuria as a result of kidney dysfunction or, more recently, several circulating microRNAs have proved their incremental value. As the number of emerging biomarkers in HFpEF is rapidly expanding, in this review, we aim to explore the most promising available biomarkers linked to key pathophysiological mechanisms in HFpEF, outlining their utility for diagnosis, risk stratification and population screening, as well as their limitations.

## 1. Introduction

**Figure 1:**
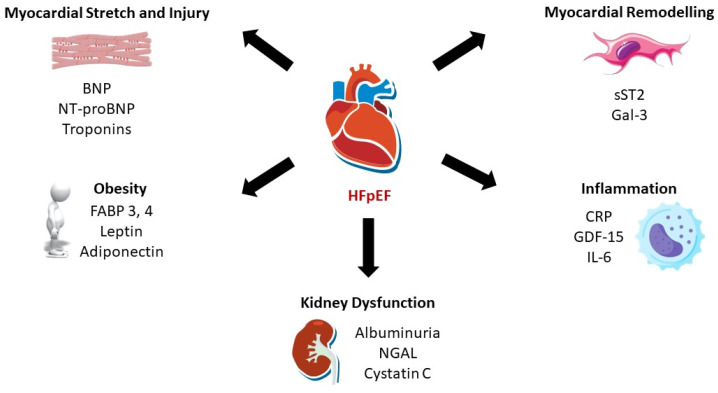
Multiple pathophysiological mechanisms involved in HFpEF and their representative biomarkers. Abbreviations: BNP, brain natriuretic peptide; NT-proBNP, N-terminal pro-B-type natriuretic peptide; FABP 3, 4, fatty acid binding protein 3, 4; NGAL, neutrophil gelatinase-associated lipocalin; CRP, C-reactive protein; GDF-15, growth differentiation factor 15; IL-6, interleukin-6; sST2, soluble suppression of tumorigenicity-2; Gal-3, galectin 3.

Heart failure (HF) is a complex clinical syndrome, resulting from a structural or functional abnormality of the heart, which leads to the classic cardinal symptoms of dyspnea, oedema and fatigue, usually accompanied by other signs, such as elevated jugular venous pressure and pulmonary crackles. At present, based on the measurement of left ventricular ejection fraction (LVEF), three distinct phenotypes are described: HF with reduced ejection fraction (HFrEF), defined as LVEF ≤ 40%; HF with mildly reduced ejection fraction (HFmrEF), with LVEF between 41% and 49%; and HF with preserved ejection fraction (HFpEF), with LVEF ≥ 50% [1]. HFpEF accounts for more than half of all cases of HF, representing one of the major public health problems worldwide [2,3]. Moreover, the diagnosis of HFpEF is often challenging, as signs and symptoms may be subtle or attributable to other comorbidities. As the incidence of this disease is continuously growing, with long-term mortality and re-hospitalization rates similar to HFrEF, major interests have risen regarding the optimal diagnostic and prognostic algorithms [4]. Ever since the concept of HFpEF as the result of a systemic proinflammatory state induced by multiple associated comorbidities emerged, major interests have focused on understanding the various pathophysiological mechanisms that lead to this complex syndrome [5]. The attempts to characterize each pathway by certain biomarkers have paved the way for a new field of research, leading to the discovery of hundreds of potential biomarkers in HFpEF. Most of the research efforts have mainly centered on HFrEF, demonstrating the incremental clinical utility of several biomarkers. The heterogeneous nature of HFpEF makes it difficult to find a single, ideal biomarker of diagnostic and prognostic value. This review aims to characterize the most promising biomarkers that demonstrated value in refining the diagnosis, risk stratification and monitoring response to treatment in patients with HFpEF (Figure 1). For this general review, we retrieved the most relevant studies from PubMed, searching for the literature in English, involving human subjects and published mainly from 2020 until 1 June 2024. We also included some older research articles that were considered relevant to the content of our review, such as landmark studies or those related to pathophysiological mechanisms in HFpEF. We excluded studies that referred only to HFrEF. Terms related to “Heart Failure with Preserved Ejection Fraction”, “Diastolic Dysfunction”, “Heart Failure, Diastolic”, “Biomarkers” and names of each specific biomarker were used for the selection of qualified studies that analyzed the prognostic and diagnostic values of circulating biomarkers, as well as their utility for treatment monitoring in specific cases.

## 2. Pathophysiological Relevance of Circulating Biomarkers

It has long been suggested that myocardial remodeling and dysfunction in HFpEF evolve from a combination of multiple risk factors and comorbidities found in these patients, such as advanced age, obesity, hypertension, diabetes mellitus, chronic kidney disease (CKD) or chronic obstructive pulmonary disease (COPD) [6,7]. The systemic proinflammatory state induced by these coexisting factors leads to alterations in the coronary microvasculature, promoting cardiomyocyte hypertrophy and increased collagen deposition [5]. Moreover, the metabolic syndrome (MetS) and each risk factor for MetS generate changes in LV geometry, leading to the development of diastolic dysfunction [8]. Incriminated pathophysiological mechanisms are cardiomyocyte apoptosis caused by lipotoxicity, increased oxidative stress determined by hyperglycemia and lipoapoptosis triggered by excessive lipid accumulation in the myocardial cells [9,10,11,12]. These changes will further result in the complex remodeling of the left ventricle characteristic of the HFpEF phenotype, which is distinct from other forms of HF. This paradigm has led to the study of an extensive array of biomarkers associated with distinct pathophysiological mechanisms in HFpEF, including myocardial remodeling, systemic inflammation, myocyte death, oxidative stress, obesity and anemia.

Currently, only the biomarkers that reflect myocardial stretch, namely the natriuretic peptides (NPs), fulfill the criteria for an ideal biomarker, although their measurement demonstrated several limitations that seriously affect their performance [13,14]. Among the biomarkers that reflect myocardial injury, cardiac troponins demonstrated strong prognostic significance in both acute and chronic HFpEF [15,16,17]. Apart from their utility in acute settings, their levels also rise in chronic cardiovascular (CV) conditions, as a consequence of a higher wall stress and impaired microvascular function in the myocardium, which makes them valuable biomarkers reflecting the severity of diastolic dysfunction [18,19]. In HFpEF, microvascular dysfunction is mediated by pro-inflammatory cytokines, leading to monocyte infiltration and differentiation into macrophages, amplifying the inflammatory response in the myocardium. Considering the biomarkers highly expressed in any state of systemic inflammation, growth differentiation factor 15 (GDF-15) showed the most promising results, identifying patients with an early stage of HFpEF and providing important prognostic information, independent of the other biomarkers [20,21,22]. Furthermore, other biomarkers play key roles in the process of myocardial remodeling by interacting with extracellular matrix proteins. Such is the case of galectin-3 (Gal-3), a protein that promotes myocyte hypertrophy and collagen deposition in HFpEF. Recent studies suggest the ability of this biomarker to identify individuals at high risk of developing HFpEF, as well as to predict adverse outcomes in both acute and chronic settings [23,24]. Similar findings are related to suppression of tumorigenicity-2 (ST2), an agent promoting inflammation and fibrosis, useful for risk stratification in HFpEF and monitoring treatment response [25,26]. Ultimately, circulating biomarkers reflecting kidney dysfunction and obesity are useful for detecting early stages of HFpEF and predicting negative outcomes in those with established disease.

Overall, the field of biomarkers is rapidly expanding and efforts are directed towards finding molecules that have both clinical and pathophysiological significance. In the following parts, we will present the clinical implications of the most promising biomarkers currently under study, with respect to their screening performance, diagnostic and prognostic ability in both acute and chronic settings and their potential usefulness for monitoring treatment response in HFpEF.

## 3. Myocardial Stretch and Injury

### 3.1. Natriuretic Peptides

The discovery of NPs laid the foundation for a revolutionary approach in patients with HF. From its initial identification in the porcine brain to a sophisticated biomarker involved in the pathophysiology of HF, brain natriuretic peptide (BNP) has demonstrated great value as a diagnostic and prognostic tool across the entire ejection fraction (EF) spectrum [27]. The NP system consists of three hormones. As the name suggests, atrial natriuretic peptide (ANP) is secreted primarily by the atria, while BNP, despite its name, is secreted by the ventricular myocardium in response to the increased myocardial wall stress [28]. C-type natriuretic peptide (CNP) is expressed at high levels in the bone, brain and vascular endothelium, with limited applicability in HF. The physiological role of NPs is related to their diuretic, natriuretic and vasodilatatory properties, acting as important regulators of blood pressure and circulating volume, thus counteracting the deleterious effects of the renin-angiotensin system (RAS) and the sympathetic nervous system. In addition, they exert anti-hypertrophic and anti-fibrotic properties in the myocardium and inhibit the proliferation of vascular smooth muscle cells [29,30]. Due to their longer plasma half-lives, BNP and its inactive metabolite N-terminal pro B-type natriuretic peptide (NT-proBNP) have been extensively studied, becoming the gold standard biomarkers in HF.

#### 3.1.1. Screening

Studies suggest that measurement of NPs may identify patients at risk of developing HF, although the cut-offs used for risk prediction in the apparently healthy population are not well established. NP levels could be used to optimize medical treatment in patients at high risk of developing HF. As the PONTIAC study demonstrated, in patients with type 2 diabetes mellitus and a NT-proBNP level > 125 pg/mL, up-titration of RAS antagonists and beta-blockers significantly reduced the primary outcome of 2-year CV mortality and hospitalizations [31]. A strategy based on BNP screening was also assessed in another randomized trial encompassing more than 1300 patients with at least one risk factor of HF or CV comorbidity, with a follow-up of 4.2 years. Results suggested that BNP is a good risk predictor for HF and CV events, as the measurement of BNP levels enhanced treatment optimization and adoption of lifestyle measurements [32]. Another recent meta-analysis suggested a reasonable diagnostic performance of NPs for the detection of diastolic dysfunction and HFpEF, at a cost of significant heterogeneity among studies [33].

#### 3.1.2. Diagnosis

The Breathing Not Properly trial was the first to evaluate the use of BNP as a diagnostic test in patients complaining of dyspnea. At a cut-off of 100 pg/mL, BNP had 90% sensitivity and 73% specificity for the diagnosis of HF [34]. Similar findings were reported from the N-terminal Pro-BNP Investigation of Dyspnea in the Emergency department (PRIDE) study, with NT-proBNP demonstrating high sensitivity and specificity for the diagnosis of acute HF at cut-offs above 450 pg/mL or 900 pg/mL for patients below or above 50 years, respectively [35]. Ever since, the measurement of NPs has become a central element in the diagnostic algorithm for acute and chronic HF, as stated in both the European and the American guidelines. Circulating levels of NPs seem to be more increased in HFrEF compared to HFpEF, although no cut-off can accurately discern between the two conditions [36]. Moreover, levels of NPs are influenced by a number of conditions and comorbidities, affecting the accuracy of HF diagnosis. NP levels become more increased in the elderly, possibly as a consequence of the decrease in renal function, so that different cut-offs should be applied according to the age group [13]. Among the comorbidities that lead to increased levels of NPs, atrial fibrillation, COPD, pulmonary hypertension (PH) and CKD are notable. These conditions are actually frequent in HF patients, affecting the diagnostic sensitivity of NPs. As a result, cut-offs for diagnosis of HFpEF need to be adjusted according to age, gender, race, ethnicity and the presence of comorbidities and on-going treatment with neprilysin inhibitors. On the contrary, obesity tends to lower NP levels, presumably attributable to an increased clearance by adipocyte NPs’ receptors, although other mechanisms are incriminated [14,37].

#### 3.1.3. Prognosis

NPs demonstrate significant prognostic implications, as demonstrated by several studies. BNP levels are strongly associated with all-cause mortality and HF hospitalizations across the entire HF phenotype [1,38]. At similar BNP levels, the prognosis of patients with HFpEF is as poor as those with HFrEF [36,39]. The same results are reported for NT-proBNP levels, which are associated with adverse events at 1-year follow-up for both HFpEF and HFrEF [40]. In the PARAGON-HF trial, NT-proBNP levels at screening were strongly associated with a risk of CV death and HF hospitalizations [41].

Changes in NP levels during hospitalizations are also important in risk stratification [1,38]. In a post hoc analysis of the SURVIVE trial, mortality at 30 and 180 days was significantly higher in the non-responders’ group, defined as patients who did not achieve a reduction in BNP level by ≥30% from baseline to day 5 [42]. Similar results regarding longitudinal changes in NP values were reported by other studies [43,44,45].

Discharge NP concentrations are also associated with clinical outcomes in HF [1]. In a sub-analysis from the OPTIMIZE-HF (Organized Program to Initiate Lifesaving Treatment in Hospitalized Patients with Heart Failure) registry, discharge BNP was a better predictor of 1-year mortality rate and rehospitalizations than admission BNP or ratio of the discharge/admission BNP [46]. Another study concluded that the absolute value of BNP at discharge was a better predictor of 6-month mortality than baseline BNP or the reduction in BNP during hospitalization [47].

#### 3.1.4. Treatment Response

Regarding the effect of treatment on outcomes across the spectrum of NP levels, a sub-analysis from the TOPCAT trial (Treatment of Preserved Cardiac Function Heart Failure with an Aldosterone Antagonist Trial) revealed a greater benefit of spironolactone in the group with lower levels of NPs as compared to higher levels [48]. The same results were reported from the I-PRESERVE trial (Irbesartan in Heart Failure with Preserved Ejection Fraction), where the angiotensin-receptor blocker significantly reduced outcomes in patients with lower levels of NPs, although treatment with irbesartan showed no benefit in the overall population of HFpEF [49]. This could be explained by the fact that higher NP levels are encountered in older patients, with more comorbidities and more advanced structural heart disease associated with HFpEF. These findings suggest that drug intervention in HFpEF might be beneficial earlier in the course of the disease. This hypothesis should be tested in specifically designed clinical trials, according to specific concentrations of NPs below which positive results become prominent. On the other hand, following an analysis from the EMPEROR-Preserved trial, treatment with empagliflozin resulted in a relative reduction in risk across all NT-proBNP concentrations, with greatest absolute reduction observed in the population with the highest concentrations [50]. Similarly, in the PARAGON-HF trial, modest overall treatment effects of sacubitril/valsartan were reported across the entire spectrum of baseline NT-proBNP. Patients who experienced the greatest reduction in NT-proBNP during treatment had better outcomes [41].

Considering an NP-guided management of patients with HFpEF, studies have been contradictory. A sub-analysis of the TIME-CHF trial (Trial of Intensified versus standard Medical therapy in Elderly patients with Congestive Heart Failure) compared a symptom-guided versus an NT-proBNP-guided therapy, revealing that the latter tended to worsen primary outcomes at 18 months [51]. Another meta-analysis confirmed that NP-guided therapy is not superior to guideline-directed therapy in acute or chronic HF, regardless of the EF [52]. Overall, evidence supporting an NP-guided strategy in HFpEF is lacking.

### 3.2. Troponins

Apart from acute coronary syndromes, troponin levels can be increased in several other conditions, such as myocarditis, pulmonary embolism or HF, particularly when measured using high-sensitivity (hs) assays. Previously, it has been hypothesized that troponins can also be released in the bloodstream in the absence of myocyte death, a term named “cytosolic pool” [19]. As a consequence, the mechanisms responsible for troponin release in HF involve both ischemic and non-ischemic processes [18,19]. The latter may be related to the increased wall stress caused by volume or pressure overload, a pro-inflammatory state caused by circulating cytokines, catecholamines and oxidative stress and impairment of diastolic function [19]. In HFpEF, troponin levels are directly correlated with LV filling pressures and diastolic dysfunction, especially during exercise [53].

#### 3.2.1. Prognosis

Several studies outlined the prognostic utility of high-sensitivity cardiac troponin T (hs-cTnT) in HFrEF [54]. In a meta-analysis including 9289 patients, the majority with HFrEF, hs-cTnT at a cut-off of 18 ng/L emerged as a strong, independent predictor of all-cause mortality (HR, 1.48; 95% CI, 1.41–1.55), CV mortality (HR, 1.40; 95% CI, 1.33–1.48) and hospitalization (HR, 1.42; 95% CI, 1.36–1.49), over a median follow-up of 2.4 years [55]. Although troponin levels are more elevated in HFrEF as compared to HFpEF, studies indicate a prognostic role for troponins in HFpEF as well [56,57].

Acute HF

In acute decompensated HF, hs-cTn levels carry important prognostic information [15,58]. In a sub-analysis of the ADHERE trial, including more than 60,000 patients, approximately 40% of patients testing positive for troponin had an EF above 40%. Compared to patients with negative troponin levels, those with detectable levels had an increased risk of in-hospital mortality (8% vs. 2.7%, *p* value < 0.001), prolonged hospitalization (adjusted mean stay 6.6 days vs. 5.5 days, *p* value < 0.001) and requirement for cardiac procedures. This association was maintained after adjustment for other established risk factors associated with negative outcomes [59]. In another observational cohort study including more than 34,000 patients with acute decompensated HFpEF, troponin elevation was significantly associated with in-hospital, 30-day and 1-year mortality and prolonged hospitalization [60].

Chronic HF

Elevated hs-cTnI levels were associated with a composite endpoint of all-cause mortality and HF rehospitalization in a cohort of patients with chronic HF, after adjustment for other established prognostic variables such as age, sex, smoking, diabetes, renal function and NT-proBNP levels. When comparing the prognostic significance across the EF, both hs-cTnI and hs-cTnT were more strongly associated with composite adverse events in HFpEF (TnI: HR 2.32; 95% CI 1.60–3.36; TnT: HR 3.01; 95% CI 2.01–4.51) than in HFrEF (TnI: HR 1.29; 95% CI 1.16–1.42; TnT: HR 1.36; 95% CI 1.22–1.53) [17].

The prognostic utility of hs-cTn was also demonstrated in several major trials in HFpEF. In a sub-analysis from the EMPEROR-Preserved Trial, similarly to NT-proBNP concentrations, hs-cTnT levels were significantly associated with the primary endpoint of CV mortality and HF hospitalizations. No treatment heterogeneity was noted, with empagliflozin demonstrating a comparable reduction in events rate across all hs-cTnT quartiles, although patients with the highest baseline hs-cTnT had the greatest absolute risk reduction. In the placebo group, patients with higher hs-cTnT values had a 4-fold increased risk for the primary endpoint [50]. Comparably, in another analysis from EMPEROR-Preserved, has-cTnT alongside NT-proBNP were the major predictors of the primary outcome (CV mortality, HF hospitalization). Those with lowest values for NT-proBNP and hs-cTnT had a primary event rate of 2.2 per 100 patient-years, in contrast with 19.2 per 100 patient-years in patients with the highest values for both biomarkers [61]. A risk model combining hs-cTnT and NPs provided important complementary information of prognosis, with good prognostic capacity (*c*-statistics ranging from 0.71 to 0.75 for mortality and the composite endpoint of HF hospitalizations or CV mortality) [61]. In another post hoc analysis from the TOPCAT trial, higher levels of hs-cTnI were independently associated with an increased risk of CV mortality or HF hospitalization (HR, 1.42; 95% CI, 1.20–1.69; *p* value < 0.001 per doubling of hs-cTnI). No treatment heterogeneity was noted across hs-cTnI levels with regard to the primary composite endpoint [16].

#### 3.2.2. Screening

The association between hs-cTn and incident HF has been insufficiently studied. In a community-based cohort of more than 8000 asymptomatic patients, no biomarker was significantly associated with an increased risk of developing HFpEF [62]. After 11.4 years follow-up, hs-cTnT and NT-proBNP were significant predictors for HFrEF and not HFpEF [63]. In a study including more than 22,000 patients, hs-cTn demonstrated a significant association with HFrEF (HR, 1.37; 95% CI, 1.29–1.46; *p* < 0.001), whereas for HFpEF, the association was also suggestive (HR, 1.11; 95% CI, 1.03–1.19; *p* = 0.008) [64]. In another retrospective analysis of the STOP-HF study, hs-cTnI at baseline and follow-up significantly predicted new-onset HFpEF [65].

## 4. Inflammation

Systemic inflammation plays a central role in the development and progression of HFpEF. Upregulation of inflammation in HFpEF leads to worse cardiac structural and functional abnormalities, demonstrated using a wide array of biomarkers. The most promising ones are illustrated in Table 1. The first trial to provide evidence supporting the relationship between inflammation and HF outcomes was the CANTOS trial, in which patients with a history of myocardial infarction and systemic inflammation were either assigned to receive an IL-1β blocker or placebo [66]. In a sub-analysis of this trial, canakinumab was found to reduce HF hospitalizations in a dose-dependent manner, generating the hypothesis that cytokine inhibition may improve HF outcomes [67].

### 4.1. GDF-15

In healthy states, GDF-15, a member of the transforming growth factor-β superfamily, is expressed at low levels by various cell types, such as cardiomyocytes, adipocytes, vascular smooth muscle cells and macrophages [79]. Overexpression of GDF-15 is triggered by inflammation, oxidative stress, tissue injury and hypoxia. As a result, this biomarker is secreted in various pathological conditions, including numerous solid cancers, metabolic conditions such as anorexia or cachexia and autoimmune diseases [79,80,81] [82,83]. In HF, the expression of GDF-15 is associated with ischemia, neurohormonal activation, pro-inflammatory cytokines and mechanical strain [84]. Concentrations of GDF-15 increase in various CV conditions, including acute and chronic HF, atrial fibrillation and acute coronary syndromes.

#### 4.1.1. Prognosis

The prognostic value of GDF-15 has been demonstrated in patients with HFrEF. In several studies, GDF-15 was shown to predict mortality and HF hospitalizations [85,86,87]. The diagnostic and prognostic utility of GDF-15 in HFpEF have also been studied in the last few years.

Acute HF

Regardless of the EF, admission GDF-15 levels were associated with all-cause mortality at 30 days in a prospective study including patients admitted for acute HF. In addition, reduction in GDF-15 levels between admission and discharge was associated with a lower rehospitalization rate [20]. In another study including patients hospitalized for acute HFpEF, GDF-15 measured within 48 h of admission independently predicted HF rehospitalization at 1 year, outperforming the prognostic value of NT-proBNP [68]. In another prospective study including 380 hospitalized patients with decompensated HFpEF, a multi-biomarker model was assessed for the ability to predict mortality at 2 years. GDF-15 was among the biomarkers independently associated with the primary endpoint, alongside NT-proBNP, hs-cTnT, TNFα and other biomarkers of extracellular matrix turnover [69].

Chronic HF

In a prospective study including 311 patients with HFpEF and HFmrEF, GDF-15 concentration but not NT-proBNP was an independent predictor of all-cause mortality, with an area under receiver operating characteristic curve (AUC) of 0.797 for the model including NT-proBNP versus an AUC of 0.819 for the overall model including GDF-15 (*p* value 0.016) [70]. In another prospective study comparing plasma GDF-15 levels in patients with HFrEF versus HFpEF, GDF-15 remained a significant predictor for the composite outcome of mortality or first HF hospitalization across both HF phenotypes. Median GDF-15 baseline values were similarly increased in both groups. When added to established clinical predictors, such as hs-cTnT and NT-proBNP, GDF-15 increased the AUC from 0.720 to 0.740 (*p* value < 0.019) [71]. In a randomized controlled trial assessing an anti-fibrotic agent versus placebo in patients with HFpEF, although no influence of treatment on changes in GDF-15 levels was noticed, changes in multiple variables were associated with an increase in GDF-15 over 1 year. These included increased NT-proBNP levels, anemia, diastolic dysfunction and right ventricular dilation, factors known to be associated with worse prognosis in HFpEF [88]. This suggests that GDF-15 may not be involved in myocardial fibrosis in patients with HFpEF but more probably with an inflammatory state. A recent meta-analysis assessed the prognostic value of GDF-15 in more than 6000 patients with chronic HF, regardless of the EF. A 6% increase in risk of all-cause mortality was noted for every 1 LnU increase in baseline GDF-15 concentration after multivariable adjustment (HR 1.06, 95% CI 1.03–1.10, *p* value < 0.001). In addition, the association of GDF-15 with all-cause mortality was more significant among ischemic HF patients [21].

Similarly to NP levels, aging increases concentrations of GDF-15 in healthy individuals [89]. However, one major distinction between these two biomarkers seems to be related to atrial fibrillation, a frequent comorbidity encountered in HFpEF. In a sub-analysis from the ARISTOTLE trial, including more than 18,000 patients with atrial fibrillation randomized to either apixaban or warfarin, GDF-15 predicted major bleeding events, stroke or systemic embolism and mortality, independently of NT-proBNP or hs-cTnI [90]. Concordantly, in a nested prospective biomarker study of the ENGAGE AF-TIMI 48 trial, including 8705 patients, elevated GDF-15 was independently associated with higher rates of stroke and major bleeding. The novel ABC-bleeding score, which included GDF-15 and hs-cTnT, outperformed the HAS-BLED score, serving to stratify patients who would benefit more from treatment with edoxaban compared to warfarin [91]. In another study of 1941 patients, comparing NT-proBNP and GDF-15 levels in patients with atrial fibrillation versus sinus rhythm, GDF-15 levels were not significantly influenced by the presence of atrial fibrillation, after adjustment for clinical confounders [92]. This would represent a major benefit for the assessment of patients with both HFpEF and atrial fibrillation, as levels of NT-proBNP are severely influenced by the presence of this arrhythmia.

#### 4.1.2. Diagnosis and Screening

A prospective study including 507 patients referred for cardiac-related symptoms, without a history of HF or other CV diseases, identified distinct phenogroups using the HFA-PEFF score. Each phenogroup was further analyzed for its association with distinct biomarkers [72]. GDF-15 was among the biomarkers that significantly differed among the four clusters, enabling us to hypothesize that distinct circulating biomarker profiles might aid in understanding the pathophysiological mechanisms of HF development and help us classify distinct HFpEF phenotypes, which could benefit from early intervention. The ability of GDF-15 to identify patients at an early stage of HF was also assessed in another prospective cohort study of more than 2000 patients. GDF-15 was the strongest predictor of HF hospitalization and all-cause mortality (HR 2.12, 95% CI 1.71 to 2.63; *p* < 0.0001), whereas a multivariable prediction model incorporating GDF-15 performed better than the one without GDF-15 [22].

### 4.2. C-Reactive Protein

Increased C-reactive protein (CRP) levels are associated with many comorbidities leading to HFpEF. As inflammation is postulated to play an important role in the pathophysiology of HFpEF, CRP could be used as a surrogate biomarker to express this state. CRP is associated with markers of HF severity, including NT-proBNP values and NYHA class, and can be used to predict negative outcomes in patients with HFpEF [73]. In a subset of patients from the TOPCAT study, hs-CRP above 2 mg/L was associated with an increased risk of adverse events, including HF hospitalizations and CV mortality. Compared to patients with low levels of hs-CRP, those with high levels had more frequent hospitalizations, an increased prevalence of COPD and a higher BMI [74]. Another meta-analysis underlined the prognostic association between higher levels of CRP and an increased risk of CV and all-cause mortality in patients with HFpEF [75].

### 4.3. Interleukin-6

As with the other biomarkers of inflammation, it has been suggested that Interleukin-6 (IL-6) could play a major role in HF. In a subset of patients from the PREVEND cohort, IL-6 was independently associated with an increased risk of developing HFpEF and not HFrEF [78]. Increased IL-6 levels are associated with symptom severity, worse renal function, poor exercise capacity and excess body fat [76]. Moreover, in patients recently hospitalized with decompensated HFpEF, IL-6 was independently associated with CV and all-cause mortality [77]. Data from the CANTOS trial suggest that the magnitude of risk reduction observed in patients receiving canakinumab was directly related to the magnitude of IL-6 reduction, with greater benefits observed in patients who achieved greater than median reductions in IL-6 [93]. Further trials will need to assess the benefits of direct inhibition of IL-6 on CV outcomes in patients with HF.

## 5. Cardiac Remodeling

Although studies have evaluated many biomarkers that reflect extracellular matrix and myocardial fibrosis, including pro-collagen propeptides, matrix metalloproteinases and tissue inhibitors of metalloproteinases, two particular biomarkers have provided the most convincing results in patients with HFpEF (Table 2).

### 5.1. Galectin-3

Gal-3 is a galactoside-binding protein from the lectin family, widely expressed in human tissues and responsible for fibroblast activation, which leads to fibrous remodeling in various organs. In the pathophysiology of HF, overexpression of Gal-3 promotes fibrosis, inflammation and cardiac remodeling, influencing progression from subclinical cardiac disease to the development of HFpEF.

#### 5.1.1. Prognosis

Acute HF

In acute HF, Gal-3 is an important prognostic biomarker. In a recent study including patients with ST-segment elevation myocardial infarction (STEMI) who underwent primary percutaneous coronary intervention, high Gal-3 concentrations were associated with HF development and rehospitalization both at 1 and 2 years [94]. Other studies support the association of Gal-3 with adverse cardiac remodeling and HF development after an acute coronary syndrome [95,96]. In a meta-analysis of 13 studies, higher serum Gal-3 levels on admission were independently associated with an increased risk of all-cause mortality (adjusted RR, 1.58; 95% CI, 1.33 to 1.88; *p* < 0.001) and CV mortality (adjusted RR, 1.29; 95% CI, 1.01 to 1.65; *p* = 0.04) in patients with acute HF, although significant heterogeneity was reported among studies [23]. Measurement of Gal-3 can also help predict short-term mortality in patients with acute HF, independently of NT-proBNP levels [97,98].

Chronic HF

The prognostic value of Gal-3 in chronic HFpEF has also been suggested. In two large cohort trials, repeated measures of Gal-3 at baseline and 3 or 6 months provided significant prognostic value. Increasing levels of Gal-3 were independently associated with all-cause mortality and HF hospitalizations [99]. A recent meta-analysis illustrated the association between Gal-3 levels and a high risk of long-term adverse outcomes in patients with HFpEF, including all-cause mortality (HR: 1.55; 95% CI: 1.27–1.87; *p* = 0.138, I^2^ = 42%), the composite of all-cause death and HF hospitalization (HR: 1.50; 95% CI: 1.30–1.74; *p* = 0.001, I^2^ = 61%) and CV death and HF hospitalizations (HR: 1.71; 95% CI: 1.51–1.94; *p* = 0.036, I^2^ = 58%) [24].

Similar to NT-proBNP, a potential link between serum levels of Gal-3 and renal dysfunction has been suggested [108]. In the RELAX trial, Gal-3 was associated with the severity of renal dysfunction but not with other pathophysiological mechanisms, assessed by biomarkers of fibrosis, inflammation or neurohormonal activation [109]. In a study including patients with acute HF, higher Gal-3 values were associated with renal dysfunction and renal tubular damage, predicting worse outcomes. After multivariable adjustment, Gal-3 remained associated with mortality during hospitalization [110]. This evidence supports the need to adjust for renal function when quantifying disease severity in HFpEF using Gal-3 levels.

#### 5.1.2. Treatment Response

The interaction between Gal-3 and response to treatment in HFpEF was also investigated. In a sub-analysis of the PARAMOUNT trial, levels of Gal-3 correlated with the severity of HFpEF, whereas baseline pre-treatment Gal-3 might have modified the response to treatment with the angiotensin receptor neprilysin inhibitor [101]. This was assessed by a reduction in left atrial volume but not in NT-proBNP, so that a definite conclusion cannot be established. Interaction between Gal-3 levels and response to spironolactone has been evaluated in the Aldo-DHF trial. Although Gal-3 levels were not influenced by treatment, increasing levels at 6 or 12 months were associated with all-cause mortality and hospitalization, independently of NT-proBNP or treatment arm [111]. Moreover, in a secondary analysis of the COACH trial, which enrolled patients with acute HF irrespective of the EF, treatment with spironolactone seemed to be more beneficial to patients with elevated Gal-3 levels, among other biomarkers [26]. These data suggest that Gal-3 is probably more useful as a diagnostic or prognostic tool, rather than a biomarker of therapy response.

#### 5.1.3. Diagnosis

The diagnostic utility of Gal-3 has been suggested in several studies, where higher levels of Gal-3 were associated with the severity of diastolic dysfunction and an increased risk of new-onset HFpEF [24,100]. Gal-3 can be used to identify individuals at risk of developing HFpEF and could be useful for phenotyping, especially in cases where fibrosis plays a major contribution to the pathology of HF. However, when comparing patients with HFpEF versus HFrEF, no significant difference is reported in serum Gal-3 levels [112].

### 5.2. Soluble ST2

Interleukin-1 receptor-like 1, commonly known in the literature as ST2, is a member of the interleukin-1 receptor family and presents as two isoforms: a soluble form—soluble ST2 (sST2)—and a transmembrane form—ST2 ligand (ST2L). Both of them bind to IL-33, which acts as either a pro- or anti-inflammatory cytokine, depending on co-stimulatory factors. The complex ST2L/IL-33 has a cardioprotective effect, limiting cardiomyocyte apoptosis, fibrosis and cardiac hypertrophy, whereas sST2 acts as a decoy receptor for IL-33, preventing its beneficial effects and leading to cardiac fibrosis, ventricular remodeling and negative cardiac outcomes [113].

Although cardiomyocytes and fibroblasts are important sources of sST2 in response to mechanical strain, in patients with HF, sST2 is secreted in large quantities from the lungs, specifically from alveolar epithelial cells. Experimental studies suggest that sST2 is involved in the pathophysiology of HF, being related to the presence and severity of pulmonary congestion [114]. sST2 measurement may offer some advantage compared to NT-proBNP, as circulating levels seem not to be affected by age, kidney function or obesity.

#### 5.2.1. Prognosis

The prognostic utility of sST2 in HFrEF, beyond NT-proBNP or hs-cTn, has been extensively studied [25,115,116]. In patients with HFpEF, sST2 is associated with pro-inflammatory comorbidities, as evidence from the RELAX trial suggests. In this trial, higher sST2 levels were significantly associated with hypertension, diabetes mellitus, atrial fibrillation, renal dysfunction, systemic congestion and right ventricular dysfunction, among others [117]. In addition, sST2 concentrations are associated with multiple echocardiographic abnormalities, including biventricular size, LVEF and RV systolic pressure [118].

Acute HF

Results from the PRIDE study revealed that concentrations of ST2 strongly predicted 1-year mortality in patients with acute HF (HR 9.3, 95% CI 1.3 to 17.8; *p* = 0.03) [102]. In the TRIUMPH cohort study, including 496 patients with acute HF, baseline ST2 was independently associated with an increased risk of the composite endpoint of all-cause mortality and HF hospitalization (per log unit, HR 1.30, 95% CI 1.08 to 1.56; *p* = 0.005). Repeated measurements of sST2 during follow-up strongly predicted outcome, regardless of NT-proBNP levels (per log unit, HR 1.85; 95% CI: 1.02 to 3.33; *p* = 0.044) [103]. In another prospective cohort study of 331 patients with acute HF, after a median follow-up of 21 months, higher sST2 levels were independently associated with CV mortality (per log unit, HR 2.174; 95% CI 1.012–4.67; *p* = 0.047) [119]. A meta-analysis including 10 studies, with a population of 4835 patients, revealed that both admission and discharge sST2 were predictive of all-cause death, CV death and the composite of all-cause death or HF hospitalization. Discharge sST2 levels were predictive of HF hospitalization during a median follow-up of 13.5 months [104].

Chronic HF

In a prospective cohort study of 193 patients, sST2 was correlated with the composite endpoint of death or HF hospitalization. This association was stronger in patients with HFpEF (per log unit, HR: 6.62, 95% CI 1.04–42.28, *p* = 0.046) compared to HFrEF (HR 3.51; 95% CI 1.05–11.69, *p* = 0.041), although median values for sST2 were lower in the HFpEF group [120]. The first meta-analysis to assess the prognostic value of sST2 in chronic HF was performed in 2017, including seven studies with a total of 6372 patients. SST2 emerged as an independent predictor for both all-cause (HR 1.75; 95% CI: 1.37 to 2.22; *p* < 0.001) and CV mortality (HR 1.79; 95% CI: 1.22 to 2.63; *p* < 0.001) in outpatients with chronic HF, regardless of the EF [105]. Further on, another meta-analysis comprising 11 studies with 5121 patients evaluated the prognostic utility of sST2 in patients with chronic HF, regardless of the EF. Increased sST2 concentrations seemed to be associated with long-term all-cause mortality (HR: 1.03; 95% CI: 1.02–1.04; *p* = 0.32, I^2^ = 0%), long-term composite of CV mortality and HF hospitalizations (HR: 2.25; 95% CI: 1.82–2.79; *p* = 0.47, I^2^ = 0%) [106]. Another recent meta-analysis revealed that elevated levels of sST2 were associated with an increased risk of the composite endpoint of all-cause mortality and HF hospitalization (per log unit, HR: 6.52; 95% CI: 2.34, 18.19; *p* = 0.985, I^2^ = 0%), after multivariable adjustment [121]. Several studies support the addition of sST2 alongside NT-proBNP, hs-cTn or other biomarkers in key pathophysiological domains for an enhanced stratification of prognosis in patients with either acute or chronic HFpEF [102,107].

#### 5.2.2. Treatment Response

Regarding the association between treatment and circulating levels of ST2, in a secondary analysis of the COACH trial, treatment with spironolactone was associated with favorable 30-day outcomes in patients with acute HF, especially in those with elevated ST-2 [26]. Data from the PARAMOUNT trial revealed that, in addition to Gal-3, sST2 was associated with the severity of HFpEF syndrome, although baseline levels of sST2 did not modify the response to Sacubitril/Valsartan. However, as in the case of Gal-3, patients with sST2 values less than the median of 33 ng/mL had a reduction in left atrial volume, which may signify a structural response to treatment [101].

#### 5.2.3. Diagnosis

The diagnostic performance of sST2 for HFpEF is overall poor when compared to NT-proBNP, although in several studies, median sST2 values were significantly higher in patients with HFpEF compared to controls [122,123]. However, since sST2 is not specific for HF, it currently has no utility in diagnosing HFpEF [121]. A meta-analysis concluded that sST2 may have some diagnostic utility in HF (sensitivity 0.72; specificity 0.65, OR 3.63; AUC 0.75), although the high heterogeneity among studies and the inclusion of case–control studies causing selection bias should be taken into account [124].

## 6. Kidney Dysfunction

### 6.1. Worsening Renal Function

Renal dysfunction is one of the most frequent comorbidities in patients with HFpEF, associated with echocardiographic and biomarker profiles of more advanced disease [125]. Worsening renal function (WRF) during hospitalizations for acute decompensated HFpEF is related to multiple pathophysiological mechanisms, such as kidney venous congestion, hypoperfusion, inflammation or treatment reactions and is associated with adverse outcomes [126]. Several studies evaluated the impact of WRF, measured by an absolute increase in serum creatinine ≥ 0.3 mg/dL, on all-cause mortality or HF rehospitalizations, proving an independent prognostic association [127,128,129].

### 6.2. Albuminuria

Albuminuria is one of the earliest markers of kidney disease, denoting underlying glomerular structural damage. It is usually quantified by measuring the urinary albumin-creatinine ratio (UACR) in a spot urine, with microalbuminuria defined as a UACR between 30 and 300 mg/g and macroalbuminuria > 300 mg/g [130]. Albuminuria is known to be associated with an increased CV risk in the general population, as well as an increased risk for CKD, regardless of other risk factors. The reciprocal pathological mechanisms of cardiac and renal dysfunction, known as cardio-renal syndromes, are illustrated by this biomarker that mirrors both diseases. Studies have documented the association between albuminuria and the development of coronary artery disease, stroke, peripheral arterial disease, microvascular dysfunction, HF and atrial fibrillation [131].

UACR is a strong predictor of adverse outcomes in HF across the entire range of LVEF [132]. In patients with HFpEF, albuminuria was independently associated with lower global longitudinal strain, increased LV and RV remodeling and worse RV systolic function. The same study demonstrated that higher UACR predicted worse outcomes in a stepwise manner across the quartiles, although the association was attenuated after adjustment for BNP levels [133]. Moreover, another recent study demonstrated that in patients with new-onset or worsening HF with both reduced and preserved EF, albuminuria was strongly associated with clinical, echocardiographic and serum markers of congestion. In addition, albuminuria independently predicted higher mortality and HF hospitalization rates [134]. In acute decompensated HF, UACR in combination with BNP levels enabled a more accurate prediction of HF rehospitalizations than BNP alone [135]. Results from the TOPCAT study revealed that albuminuria was independently associated with worse CV outcomes, while treatment with spironolactone significantly reduced UACR at 1-year follow-up compared with placebo. A reduction in UACR by 50% was independently associated with a reduction in adverse outcomes [136]. Another prespecified analysis of the FIGARO-DKD trial suggested that albuminuria screening and early initiation of treatment with finerenone in patients with CKD and type 2 diabetes reduced the incidence of new-onset HF [137]. Overall, current evidence suggests that albuminuria in patients with HFpEF portends adverse prognosis, although the underlying mechanisms are incompletely elucidated. Incorporating UACR measurement in clinical practice is useful for an enhanced risk stratification and probably for monitoring treatment efficacy, although more trials are needed to assess the influence of therapy, especially sodium glucose cotransporter-2 (SGLT2) inhibitors, on albuminuria in patients with HFpEF.

### 6.3. NGAL

Neutrophil gelatinase-associated lipocalin (NGAL) is a predictor of acute kidney injury (AKI), as its plasma and urine levels rise before an increase in creatinine becomes apparent [138]. Its potential role in the prognostic stratification of patients with HF became a subject of interest in the past few years. However, evidence supporting its prognostic value is not conclusive. One study investigating the prognostic role of urinary NGAL (uNGAL) in patients with acute decompensated HF revealed that an elevated level of uNGAL on the first day of admission was independently associated with the primary endpoint (all-cause mortality, CV death and HF readmission) and with the development of AKI [139].

In another prospective cohort study including 927 patients hospitalized with acute HF, admission and peak values of serum NGAL (sNGAL), uNGAL, uNGAL/urine creatinine ratio were compared to admission and peak serum creatinine. Other studies support the prognostic value of NGAL in acute HF [140,141]. However, neither was superior to serum creatinine in predicting the composite endpoint of mortality, HF rehospitalization or initiation of renal replacement therapy [142]. A sub-analysis from the TOPCAT trial revealed that, although NGAL tended to predict mortality or HF hospitalizations, after multivariable correction, it did not meet significance [107]. A recent meta-analysis revealed that elevated sNGAL was associated with higher mortality or the composite outcome of mortality and rehospitalizations in patients with HF [143].

### 6.4. Cystatin C

Cystatin C (CysC) is a valuable alternative marker used for estimating kidney function, as represented by GFR. Its limited relationship to muscle mass and diet confers CysC an advantage over serum creatinine. In HFpEF, several studies suggest a potential role of CysC in risk stratification. The estimated GFR using CysC is significantly associated with worse diastolic function and adverse outcomes [144]. Serum CysC on admission is a strong predictor of all-cause mortality and HF readmission at 1 year, independently of NT-proBNP [145]. The same results are confirmed in a recent meta-analysis, demonstrating that CysC is an independent predictor of adverse outcomes in patients with HF, in addition to sNGAL [146]. With a clearance entirely dependent on GFR, CysC may be a good prognostic biomarker in both acute and chronic HFpEF, superior to creatinine.

## 7. Obesity

### 7.1. Fatty Acid Binding Protein 3 and 4

Fatty Acid Binding Protein 4 (FABP-4), a lipid chaperone found in adipocytes, appears to exert cardio-depressant effects, potentially leading to systolic dysfunction in obese patients [147]. A possible pathophysiological link exists between FABP-4 and HFpEF, as it is a marker of high metabolic risk, contributing to endothelial dysfunction through a pro-inflammatory cascade [148]. FABP-4 can also predict negative outcomes in patients with HFpEF. A prospective study revealed that in HFpEF, FABP-4 levels are associated with parameters of cardiac remodeling, diastolic and systolic dysfunction, predicting all-cause mortality or HF hospitalizations during a mean follow-up of 9.1 months [149]. Fatty Acid Binding Protein 3 (FABP-3) is another cytoplasmic protein found predominantly in the heart, with a crucial role in cardiac lipid transportation in myocardial metabolism. FABP-3 can also be used as a prognostic biomarker in HFpEF, with serum levels being independently associated with subsequent CV events [150,151]. In addition, both FABP-3 and FABP-4 were associated with all-cause and CV mortality in a group of patients with chronic HF and associated type 2 diabetes mellitus [152].

### 7.2. Leptin

Leptin is excreted from the adipose tissue and is involved in modulating food intake. It has complex CV effects, protecting against LV hypertrophy, promoting weight reduction and thus acting against the development of HF [153]. The role of leptin in the development of HF is intriguing, as it is associated with both protective and risk factors [154]. It has been suggested that increased leptin levels are associated with better outcomes in HFrEF but not in HFpEF, according to one study [155]. However, in another cross-sectional study including black women with preserved EF, higher leptin levels were associated with lower myocardial stiffness and LV mass index in obese patients, conferring a possible protective effect against the development of HFpEF [156]. These results need to be further confirmed in larger studies, taking into account the variability of racial or ethnic populations.

### 7.3. Adiponectin

Adiponectin is an anti-inflammatory cytokine derived from the adipose tissue, exerting cardio-protective effects via increasing insulin sensitivity and lipid regulation. This “rescue hormone” exerts anti-apoptotic, antioxidant and anti-fibrotic effects, protecting against the development of CV disease. The role of adiponectin in HF is controversial. In HFrEF, increased levels are paradoxically associated with the severity of the disease, as well as a higher NYHA class, probably related to adiponectin resistance in the myocardium. Patients with increased concentrations of adiponectin have a poor prognosis and a higher risk of mortality, particularly those with reduced muscle mass and cachexia [157,158]. In patients with chronic HF, baseline adiponectin levels are associated with mortality, while increasing levels over 3 months are associated with worse outcomes than stable levels [159]. In contrast, low levels of adiponectin are associated with the obesity-HFpEF phenotype, especially in women [160]. In a preclinical model of hypertension-related HFpEF, low levels of adiponectin exacerbated cardiac remodeling, diastolic dysfunction and pulmonary congestion [161].

## 8. Other Biomarkers

### 8.1. Antigen Carbohydrate 125

Antigen Carbohydrate 125 (CA 125) is a plasma biomarker traditionally used for the evaluation, risk stratification and monitoring of patients with ovarian cancer. Elevated levels of CA 125 are identified in other non-malignant conditions, such as pulmonary diseases, cirrhosis or HFCARDI. Increasing evidence has emerged, suggesting that CA 125 can serve as a prognostic tool in the risk stratification of patients with both acute and chronic HF [162]. In HFpEF, evidence supports the potential value of CA 125 as a biomarker for the prediction of mortality and HF readmissions, outperforming the prognostic value of NT-proBNP in some studies [163,164,165]. A positive correlation between CA 125 levels and the presence of fluid overload, such as serous effusions and peripheral edema, has been described, making this glycoprotein a useful biomarker for extravascular congestion in HF [166]. Concentrations of CA 125 are also increased in inflammatory states, in association with different types of cytokines [167]. As a result, CA 125 correlates with parameters of disease severity and it can be useful in guiding decongestion therapy [168,169]. In contrast, a recent sub-analysis of the EMPEROR trials revealed that in patients without clinical evidence of congestion, CA 125 predicted the primary endpoint only in those with HFrEF and not among those with HFpEF. The beneficial effect of empagliflozin seemed to be attenuated in patients with lower baseline CA 125 levels [170]. In addition, the highest baseline CA 125 levels were independently associated with an increased rate of kidney function decline [171]. Future trials should focus more on the association of treatment with SGLT2 inhibitors and CA 125 levels in patients with HFpEF, as this was already suggested in those with HFrEF [172,173].

### 8.2. Iron Deficiency

Iron deficiency (ID), defined as ferritin < 100 or transferrin saturation < 20%, is highly prevalent in patients with HFpEF. Contributing factors include decreased iron absorption due to congestion, reduced availability of stored iron and nutritional deficiency. ID can impact exercise performance in patients with HF, affecting oxygen consumption leading to anaerobic metabolism. In a retrospective study of 212 patients, both anemia and ID, along with advanced age and CKD, were independently associated with all-cause mortality in HFpEF. Patients with ID expressed more severe HF symptoms and worse functional capacity compared to those without ID [174]. ID was associated with worse functional outcomes but not HF hospitalization or mortality in another meta-analysis [175]. The importance of correcting ID is currently not established in HFpEF, as opposed to HFrEF, and further trials are needed in this field.

### 8.3. Circulating microRNAs

The discovery of microRNAs (miRNAs) has opened a new door in the era of molecular biology. They represent small, non-coding, regulatory RNA molecules of 22 nucleotides that operate at the level of gene expression by targeting specific regions of messenger RNAs (mRNAs). They have been shown to be promising diagnostic and prognostic biomarkers for many diseases, including HF [176,177]. Distinct miRNAs have been associated with different pathophysiological pathways in HF. MiR-126 has major roles in maintaining endothelial homeostasis and decreased levels have been associated with microvascular endothelial dysfunction. Other miRNAs, such as miR-802 and miR-103/107, are involved in insulin sensitivity in models of diabetes and obesity [178]. In a recent case–control study including symptomatic patients with HF, 13 different miRNAs were differentially expressed in HFpEF compared to HFrEF, most of them being down-regulated, such as miR-21-5p, miR-20a-5p, miR-130a-3p, miR-103a-3p, miR-423-5p, miR-19b-3p, miR-301-3p, let-7d-5p, miR-335-5p, miR-128a-3p and miR-25-3p. These miRNAs correlated with echocardiographic and cardiac magnetic resonance imaging (MRI) parameters of HF, suggesting that miRNAs could be involved in myocardial remodeling [179]. Overall, the different miRNA profiles expressed in HFpEF and HFrEF support the different pathobiological mechanisms underlying these two entities. More research is needed in order to select specific circulating miRNAs to better serve as prognostic and diagnostic biomarkers of HFpEF.

### 8.4. Proteomics and Metabolomics

Proteomic profiling is a powerful tool that allows for a large-scale characterization of the entire protein phenotype in HF. Investigating the various patterns of proteome changes provides further evidence of the different pathogenic mechanisms involved in HF. A recent study identified 29 unique HFpEF-associated proteins related to remodeling, inflammation, fibrosis, kidney injury and lipid metabolism. Of those, AOC3, CLSTN2, Gal-9 and MATN2 were associated with HFpEF hospitalization, while 11 others, including CDH2, CSTB, KIM1, PARP-1 and SPINT2, were associated with all-cause mortality, independently of other clinical factors and NT-proBNP [180]. Another study identified novel proteins related to HFpEF subtypes, associated with platelet degranulation and microvascular dysfunction (e.g., Gal3bp, ITIH3 and von Willebrand factor) and angiogenesis (e.g., LRG1 and IGFALS) [181]. An advanced proteomic profile has the potential for establishing more precise diagnostic and prognostic CV care for patients with HFpEF.

Metabolic dysfunction plays an important role in the development of HFpEF. Patients with HFpEF display distinct metabolic profiles when compared to those with HFrEF, including markers of impaired lipid metabolism, increased oxidative stress and enhanced collagen synthesis [182]. As an example, in spite of a higher prevalence of obesity and diabetes in patients with HFpEF compared to HFrEF, metabolites of fatty acid oxidation were expressed in a lower proportion in the HFpEF myocardium [183]. Other biomarkers of interest reflecting different metabolomic pathways in HFpEF are low levels of serine, cAMP and lysophosphatidylcholine and high levels of cystine, kynurenine and acylcarnitine, among many others [184].

## 9. Future Directions

HFpEF represents a multisystem disorder, a combination of risk factors, cardiac and extra-cardiac mechanisms, which are expressed by specific circulating biomarkers. Regarding their diagnostic value, a comprehensive overview of current studies assessing novel biomarkers demonstrated a high risk of bias, impacting the reliability of their results and their clinical utility. The main study limitations referred to the use of a case–control design, exclusion of important subsets of the HFpEF population, the absence of external validation and the absence of reference standard tests to confirm the diagnosis of HFpEF [185]. In order to correctly assess the incremental diagnostic value of novel biomarkers and to ensure their clinical uptake in our daily practice, methodological well-designed studies are needed. Such trials should have a prospective design and enroll a large number of patients with HFpEF in order to cover the phenotypical heterogeneity of this syndrome.

Regarding their prognostic utility, the combination of several biomarkers encompassing different pathophysiological pathways of HFpEF may demonstrate better predictive value than using individual biomarkers alone. Several studies aimed to assess the utility of a multi-marker approach for a better risk stratification of HFpEF patients, but more research is needed in this field in order to find a predictive model that effectively discriminates for both mortality and morbidity in HFpEF.

The advent of advanced proteomics and metabolomics technologies has enabled the identification of distinct biomarker profiles in HFpEF as compared to HFrEF. Development of these molecular biology techniques allowed us to define specific “biomarker signatures” of the different HFpEF phenotypes, providing the opportunity to explore new pathophysiological mechanisms and explore the dynamic changes in biomarkers after treatment interventions. The potential of these novel biomarkers is promising, but external validation in large cohort studies is needed before using them in clinical practice.

## 10. Discussion

The burden of HFpEF is continuously growing, surpassing the prevalence of HFrEF over the past decades. As a result, defining a standardized diagnostic and prognostic approach becomes crucial. Currently, the diagnosis of HFpEF in the non-acute setting remains challenging, as patients may experience symptoms only during exertion. The current diagnostic algorithm proposed by the European Society of Cardiology performs well in diagnosing HFpEF, but many cases require costly, invasive tests to confirm the diagnosis, which may not be readily available in all centers [186]. Consequently, research efforts have lately focused on finding novel circulating biomarkers for the detection and risk stratification of HFpEF.

Currently, NPs remain the gold standard for the diagnosis and prognosis of HFpEF, although their concentrations are greatly impacted by many co-existing conditions. In addition, NPs reflect only one pathophysiological mechanism, which is insufficient to characterize the complex phenotypical spectrum of HFpEF. As stated above, myocardial dysfunction in HFpEF is the result of the interaction between CV, metabolic, renal, pulmonary and geriatric conditions, each in different proportions. The impact of systemic inflammation can be evaluated by measuring various biomarkers, among which GDF-15 demonstrated additional value in refining the diagnosis and prognosis of patients with HFpEF. In addition, biomarkers reflecting myocardial fibrosis, such as Gal-3 and sST2, as well as those reflecting obesity and CKD, are involved in the development and progression of HFpEF and can be used for optimal risk stratification.

Previous studies characterized distinct phenogroups of patients with HFpEF, with important differences in circulating biomarkers [72,187]. Creating distinct biomarker profiles in patients with HFpEF will also enable us to select those who may benefit from distinct targeted interventions, such as mineralocorticoid receptor antagonists, angiotensin-converting enzyme inhibitors or angiotensin receptor blockers [188]. Until recently, the development of effective drugs for the treatment of HFpEF has been unsatisfying, with SGLT2 inhibitors being the only drugs with demonstrated improvement in clinical outcomes. However, depending on the phenogroup, biomarkers may be used to initiate tailored medication and monitor treatment efficacy in the population of HFpEF. As a result, biomarkers in HFpEF represent a continuously expanding field, with research efforts guided towards finding the ones with additional screening, diagnostic, prognostic and therapeutic roles.

## 11. Conclusions

The pathophysiology of underlying HFpEF remains a subject of controversy, although it is likely driven by a combination of several comorbidities, leading to low-grade systemic inflammation, microvascular dysfunction and ultimately cardiac remodeling and fibrosis. Given that HFpEF presents with clinical heterogeneity, the study of different biomarkers reflecting distinct pathophysiological pathways could provide major insights into our understanding of this complex disease. Many biomarkers have demonstrated their diagnostic and prognostic utility, but their lack of specificity, high costs and the absence of standardized measurement techniques have limited their applicability in clinical practice. A strategy based not only on NPs but also on other cardiac and non-cardiac biomarkers reflecting the comorbidities of HFpEF is likely useful to define the different phenotypes, stratify risk for adverse events and monitor treatment efficacy.

## Figures and Tables

**Table 1:** Summary of biomarker studies reflecting inflammation and their clinical utility in HFpEF.

Biomarker	Application to HFpEF		
GDF-15	Prognosis	Acute HF ◦ Admission levels associated with 30-day all-cause mortality [20] ◦ Levels measured in the first 48h predict HF rehospitalization at 1 year [68] ◦ In a multi-marker strategy, predicts all-cause long-term mortality [69]	Chronic HF ◦ Predicts all-cause mortality, outperforming NT-proBNP [70] ◦ Predicts the composite of mortality or first HF hospitalization regardless of the EF [71] ◦ Associated with all-cause mortality, especially in ischemic patients [21]
	Diagnosis and Screening	◦ Levels significantly differed among 4 phenogroups of subjects with HF-like symptoms [72] ◦ Ability to identify early stages of HFpEF [22]	
CRP	Prognosis	◦ Associated with markers of HFpEF severity [73] ◦ Levels > 2 mg/dL associated with HF hospitalizations and CV mortality [74] ◦ High levels predict all-cause and CV mortality [75]	
IL-6	Prognosis	◦ Associated with HFpEF severity [76] ◦ Predicts all-cause and CV mortality in acute decompensated HFpEF [77]	
	Diagnosis	◦ Levels associated with increased risk to develop HFpEF and not HFrEF [78]	

Abbreviations: GDF-15, growth differentiation factor 15; CRP, C-reactive protein; IL-6, interleukin-6; NT-proBNP, N-terminal pro-B-type natriuretic peptide; HF, heart failure; HFpEF, heart failure with preserved ejection fraction; HFrEF, heart failure with reduced ejection fraction; EF, ejection fraction; CV, cardiovascular.

**Table 2:** Summary of biomarker studies reflecting myocardial remodeling and their clinical utility in HFpEF.

Biomarker	Application to HFpEF		
Gal-3	Prognosis	Acute HF ◦ After an ACS, high levels predict HF development and rehospitalization [94,95,96] ◦ High levels on admission predict all-cause mortality [23,97,98]	Chronic HF ◦ Repeated measures at baseline, 3 and 6 months associated with all-cause mortality and HF hospitalizations [99] ◦ Levels associated with long-term adverse outcomes: CV and all-cause mortality, HF hospitalizations [24]
	Diagnosis	◦ High levels associated with the severity of diastolic dysfunction and an increased risk of new-onset HFpEF [24,100]	
	Treatment response	◦ Baseline pre-treatment levels could modify response to treatment with ARNI, assessed by a reduction in left atrial volume [101] ◦ Possible benefit for patients with high levels, treated with spironolactone [26]	
sST-2	Prognosis	Acute HF ◦ Can predict all-cause mortality at 1 year [102] ◦ Repeated measurements during follow-up predict all-cause mortality and HF hospitalization, regardless of NT-proBNP [103] ◦ Admission and discharge levels predict negative outcomes [104]	Chronic HF ◦ Independent predictor of all-cause and CV mortality [105] ◦ Association with long-term all-cause mortality, CV mortality and HF hospitalizations [106] ◦ Enhanced risk stratification when added to NT-proBNP, hs-cTn and other biomarkers [102,107]
	Treatment response	◦ Treatment with spironolactone is associated with better 30-day outcomes in patients with acute HF and increased levels of ST-2 [26] ◦ For patients with sST-2 < 33 ng/mL, treatment with ARNI leads to a reduction in left atrial volume [101]	

Abbreviations: Gal-3, galectin 3; sST2, soluble suppression of tumorigenicity-2; hs-cTn, high-sensitivity cardiac troponin; ACS, acute coronary syndrome; ARNI, angiotensin receptor neprilysin inhibitor.

## Data Availability

The original contributions presented in the study are included in the article, further inquiries can be directed to the corresponding author.
